# Effects of Gene Dose, Chromatin, and Network Topology on Expression in *Drosophila melanogaster*

**DOI:** 10.1371/journal.pgen.1006295

**Published:** 2016-09-06

**Authors:** Hangnoh Lee, Dong-Yeon Cho, Cale Whitworth, Robert Eisman, Melissa Phelps, John Roote, Thomas Kaufman, Kevin Cook, Steven Russell, Teresa Przytycka, Brian Oliver

**Affiliations:** 1 Section of Developmental Genomics, Laboratory of Cellular and Developmental Biology, National Institute of Diabetes and Kidney and Digestive Diseases, National Institutes of Health, Bethesda, Maryland, United States of America; 2 Computational Biology Branch, National Center for Biotechnology Information, National Library of Medicine, National Institutes of Health, Bethesda, Maryland, United States of America; 3 Department of Biology, Indiana University, Bloomington, Indiana, United States of America; 4 Department of Genetics and Cambridge Systems Biology Centre, University of Cambridge, Cambridge, United Kingdom; Stanford University School of Medicine, UNITED STATES

## Abstract

Deletions, commonly referred to as deficiencies by *Drosophila* geneticists, are valuable tools for mapping genes and for genetic pathway discovery via dose-dependent suppressor and enhancer screens. More recently, it has become clear that deviations from normal gene dosage are associated with multiple disorders in a range of species including humans. While we are beginning to understand some of the transcriptional effects brought about by gene dosage changes and the chromosome rearrangement breakpoints associated with them, much of this work relies on isolated examples. We have systematically examined deficiencies of the left arm of chromosome 2 and characterize gene-by-gene dosage responses that vary from collapsed expression through modest partial dosage compensation to full or even over compensation. We found negligible long-range effects of creating novel chromosome domains at deletion breakpoints, suggesting that cases of gene regulation due to altered nuclear architecture are rare. These rare cases include *trans* de-repression when deficiencies delete chromatin characterized as repressive in other studies. Generally, effects of breakpoints on expression are promoter proximal (~100bp) or in the gene body. Effects of deficiencies genome-wide are in genes with regulatory relationships to genes within the deleted segments, highlighting the subtle expression network defects in these sensitized genetic backgrounds.

## Author Summary

Deletions alter gene dose in heterozygotes and bring distant regions of the genome into juxtaposition. We find that the transcriptional dose response is generally varied, gene-specific and coherently propagates into gene expression regulatory networks. Analysis of expression profiles of deletion heterozygotes indicates that distinct genetic pathways are weakened in adult flies bearing different deletions, even-though they show minimal or no overt phenotypes. While there are exceptions, breakpoints have a minimal effect on gene expression of flanking genes, despite the fact that different regions of the genome are brought into contact and that important elements such as insulators are deleted. These data suggest that there is little effect of nuclear architecture and long-range enhancer and/or silencer promoter contact on gene expression in the compact *Drosophila* genome.

## Introduction

*Deficiency* (*Df*) is a genetic definition for mutations that affect contiguous loci on a chromosome [1]. They are now known to be a result of DNA deletion [2] and have many important uses in genetic analysis. *Dfs* are part of an important series of tests for defining the nature of mutant alleles according to Muller's morphs [3] where, for example, an allele is said to be an amorph when, in the homozygous condition, it exhibits the same phenotype as when uncovered by a *Df* encompassing the locus. Genetic mapping by complementation tests using a series of defined *Dfs* is also common, although not necessarily definitive, since dose dependent interactions between loci (non-allelic non-complementation) can also result in mutant phenotypes [2]. Many dominant dose-dependent suppressor and enhancer mutations had already been identified in *Drosophila* by the 1930's [4] and screens for non-allelic modifiers of mutant phenotypes are one of the most important uses for large collections of *Dfs* that tile the genome. The genetic interactions uncovered in such screens can be extremely informative, since gene pairs showing dose-dependent interactions often encode near neighbors in genetic pathways or subunits of the same protein complex. "*Df* kit" screens for modifiers of a gene of interest can thus rapidly identify regions where genes encoding members of the same pathway reside [5]. However, despite the undisputed utility of *Dfs*, we know relatively little about how these widely used tools impact the transcriptome.

*Drosophila* shows very little haploinsufficiency [2], with most mutant alleles recessive to the wild type allele. The largest group of haploinsufficient loci is the *Minutes*, which encode ribosomal proteins or elongation factors [6], suggesting that there is a very strong requirement for diploidy when it comes to ribosome biogenesis. However, like many other animals, *Drosophila* is sensitive to large-scale reduction in gene dose. In a classic study, the entire genome was examined for dosage effects using a set of crosses between translocation-bearing flies [7] and this segmental aneuploidy screen demonstrated that, outside of haploinsufficient regions, deleterious effects of gene dose reduction are generally dependent on the amount of material removed rather than the particular locus. This pioneering work suggested that there are many small additive or cumulative effects of reduced gene dose and, as the extent of a deleted segment grows, more genes in any given pathway are perturbed [8]. Thus, it appears that the effects of dose alteration accumulate, propagate, and eventually collapse gene networks. The observation that Drosophila can tolerate deletions of up to approximately 1% of the euchromatic genome [7] is likely to reflect the connectivity of the gene network and the limits of network robustness [8]. The small effects associated with dose reduction are the main reason that *Dfs* are so useful in enhancer and suppressor screens. The dose changes in pairs of genes close in a network result in a phenotype, even though dose reduction of either alone is without overt consequence.

With the more recent application of genomic approaches, we are beginning to understand more about the effect of gene dose on the expression of autosomal hemizygous (one copy) genes in *Drosophila*. Gene expression does go down when gene dose is reduced, but not by 2-fold [8–16]. Genes tend to be expressed at a higher level than expected if there is a simple one-to-one relationship between copy number and expression level. This topic is relevant to a host of systems and is not without controversy, as the deviation from expected values are small in experiments with several diverse organisms. For example, small naturally occurring copy number changes in yeast show gene-by-gene compensation, while engineered whole chromosome aneuploids show a stress response but little evidence of compensation [17, 18]. The differences in conclusions may be due to the regions deleted. Whole chromosome aneuploids in *Drosophila* include many genes encoding the highly dose-sensitive ribosomal proteins. A study with highly aneuploid human HeLa cell lines demonstrated modest compensation (~ 1.2 fold, [19]) and many highly aneuploid *Drosophila* cell lines also show compensation [15, 16]. Similarly, human population-based studies show partial to almost full transcriptional compensation at genes showing copy number variation [20–22]. In trisomies, 10 to 30% reduction in trisomic gene expression has been seen for both human trisomy 13 and 21 in primary cells [23]. It seems clear that aneuploidy alters gene expression that does not often scale perfectly with gene dose. The question is how important these subtle changes are, and what are the mechanisms that result in specific dosage responses.

There has been debate on whether the non-sex-chromosome (autosomal) dosage compensation response in *Drosophila* is due to a general effect, elevating the expression of all hemizygous genes, or to a gene-by-gene effect consistent with classic gene regulation [8, 10]. These are not mutually exclusive models. Modest, but measurable, autosomal dosage compensation in *Drosophila* could be due to the biochemical properties of pathways such as flux, and the regulatory interactions such as feedback, commonly found in molecular biology [24]. Alternatively, a more global response to aneuploidy that specifically recognizes aneuploid segments and increases expression of all genes in that segment could operate [12]. The latter is analogous to the sex chromosome dosage compensation system that globally increases expression of the single *X* in wild type *Drosophila* males [25]. Within the genome there are many genes that show a consistent and modest dose response, which could be due to a general buffering system like flux, but there are also dramatic outliers, where expression of one copy genes collapses or actually increases (the inverse effect [26]). The extreme cases are more consistent with disrupted positive or negative feedback loops. However, the best evidence for gene-by-gene regulatory compensation is the coherent propagation of expression changes across gene expression networks observed in *Df/+* flies [8].

One way to help address issues relating to mechanisms of autosomal dosage compensation would be to obtain a larger sample of expression profiled *Df* lines to see if responses could be classified. We have therefore examined the effects of chromosome arm *2L Dfs* [*Df(2L)*] on transcription in adult females and males in two genetic backgrounds, generating a total of 813 expression profiles in biological duplicate (or greater). We show that dose-dependent gene expression is generally locus-specific. Some genes are perfectly compensated, some genes show no evidence of compensation, the majority are somewhere in between, and a few dramatic outliers show collapsed expression or greatly elevated expression. Transcription is an enzymatic process. We suggest that pathway flux and kinetics (buffering) and molecular regulatory circuits (feedback) for a given gene accounts for most autosomal dosage responses and compensation [27]. However, we also find that there are regional responses for a minority of genes. The genome is organized into chromatin domains flanked by insulators [28] and we provide evidence that deletions within chromatin domains associated with repressive chromatin marks result in superior compensation or even over-expression in females. This suggests that there is a *trans* effect of *Dfs* that can weaken repressive domains.

Because genes function in networks, changes in gene expression in one copy regions should alter the expression of genes in the same pathway due to regulatory interactions. We find strong support for this type of network structure in the expression profiles, since we observed that reduction in transcript levels from one copy genes propagates to primary network neighbors. These networks do not collapse completely, as the flies in question are viable and fertile. However, we can track expression change through the network. Near neighbors in the regulatory network showed the greatest disruption, which dissipated with increasing separation in the network. We also observe more loosely connected (and often dramatic) changes that may represent pathway compensation. Our results suggest that we can learn much about the logic of gene networks by measuring how they respond to dose changes in hemizygous conditions without overt phenotypes, rather than profiling mutants with morphological, physiological, or behavioral phenotypes that complicate pathway analysis.

We report on two additional aspects of the effect of *Df(2L)s* on the transcriptome. First, *Df* breakpoints bring together two regions of the genome that are usually distant in the linear chromosome. This can result in breakpoint proximal changes due to transcription unit fusions, or local changes due to juxtaposition of regulatory regions such as enhancers. If long-range promoter/enhancer interactions are common [29], or if chromatin domains with blocks of repressive or active chromatin spread when insulators are deleted (as in [30]), then one would expect altered expression within novel chromatin or regulatory domains flanking breakpoints. Somewhat surprisingly, but in agreement with previous work on *Drosophila* inversions [31], our results suggest that there is very little functional long-range promoter communication with enhancers or silencers, and that disrupting chromatin domains is generally innocuous in terms of transcription. However, as in the cases of dosage compensation due to de-repression, there are exceptions, where cis-regulatory domains appear to have been deleted or fusion of chromatin domains appears to result in spreading effects on transcription.

## Results

**Fig 1:**
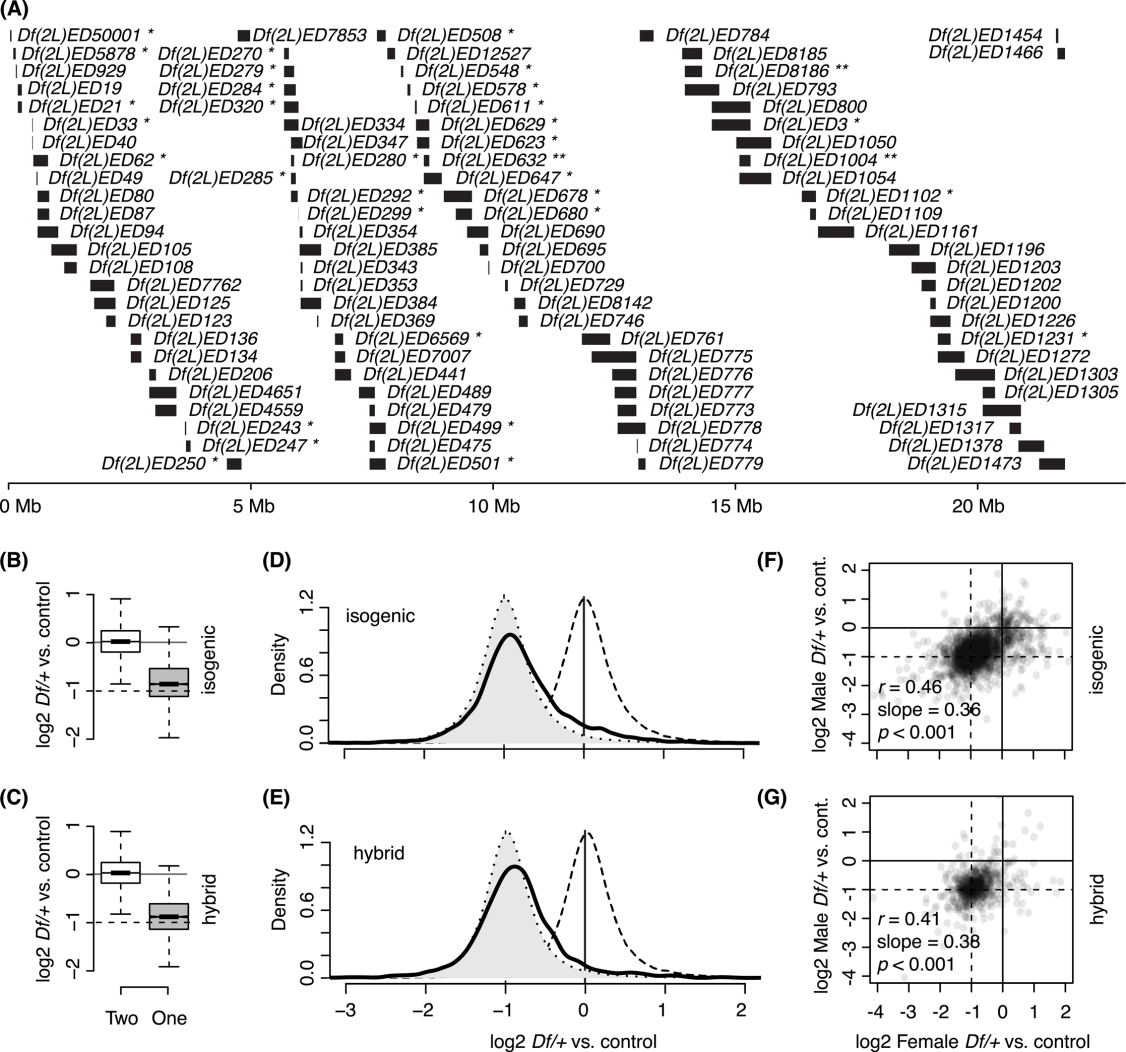
Transcriptional responses to gene copy on Chromosome 2L. A) *Dfs* used in this study. The extent of the deleted DNA (bars) and position along the first 25Mb of *2L* (bottom scale) are indicated. *Dfs* were tested in both the isogenic and hybrid background (except: * isogenic only, and ** hybrid only). B, C) Box plots of gene expression in two copy genes (open) and one copy genes (filled) relative to normalized global expression of the same genes in the rest of the dataset. Bottom, middle, and top lines of each box represent the 1^st^, 2^nd^ (median), and 3^rd^ quartile of the distribution, respectively. The maximum or minimum observation within 1.5 times of the interquartile range (3^rd^ quartile– 1^st^ quartile) from the 3^rd^ or 1^st^ quartile, respectively is indicated by whiskers. Notches indicate the 95% confidence interval for the medians. A 2-fold difference in expression is delimited by the solid and dotted horizontal lines. D, E) Normalized relative expression value distributions of two copy genes (dashed line, open), the projected distribution if gene expression was reduced by 50% in one copy genes (dotted line, filled), and observed one copy gene expression (solid line, open). F, G) Scatter plots that display one copy gene expression levels between males and females from same *Dfs*. Pearson’s correlation coefficient (*r*), regression slope, and *p* value for the correlation are indicated. B, D, F) Isogenic background. C, E, G) Hybrid background.

To systematically investigate the effects of deletions on transcription, we expression profiled a set of molecularly defined hemizygous DrosDel fly lines [32] uncovering approximately 68% of the euchromatic portion of the left arm of chromosome *2* (*2L*; **Fig 1A**). We examined gene expression in adult females and males from 99 different *Df* lines following backcrosses to the *w*^*1118*^ parental line used to generate the DrosDel collection. In practical applications of *Df* screening, *Df* lines are typically outcrossed to other genetic backgrounds to map mutations or look for dose-dependent genetic interactions. To determine how sensitive dosage responses were to genetic background, environment, and other factors that may differ in "replicated" experiments between labs, we also examined adult female and male gene expression in 67 of these *Df* lines in a hybrid background by outcrossing to the sequenced modENCODE *OregonR* line [15] for a total of 102 expression profiled *Dfs*. These hybrid background flies were also reared in different laboratories on different media. This cross provides a strong test of the robustness of perturbations we report. For the expression profiling, we performed multiplexed RNA-Seq on polyA^+^ selected RNA, used ERCC spike-in controls [33] from either single flies or pools to characterize measurement variance and ratiometric performance, and determined low expression cutoffs based on an evaluation of intergenic expression. At a minimum, we used biological duplicates for each *Df* and each sex for a total of 813 expression profiles, which are available in the Gene Expression Omnibus (GEO [34], accessions GSE61509 and GSE73920).

### Gene dose responses

To compare one copy expression to two copy expression for individual genes on *2L* we took advantage of the fact that there were many *Df/+* lines where a given gene was two copy. Therefore, for any given gene, we took the two copy gene expression in all other lines as a reference for the expression when that gene was only in one copy. To summarize the typical responses of genes to their own dose, we pooled the data for one copy gene expression across all *Df/+* genotypes within the isogenic or hybrid backgrounds. In both genetic backgrounds, we observed a clear reduction in gene expression from one copy (*p* < 0.001, Mann Whitney U test) as compared to two copies. However, our analysis confirmed previous reports that reduced expression is not 2-fold [8, 12–14]. We observed a mean 1.1-fold compensation (*p* < 0.001) against gene dose reduction (**Fig 1B and 1C**). As in previous work, we observed that compensation was not due to a uniform effect on all genes, as one copy gene expression was skewed towards compensation (**Fig 1D and 1E**; Pearson’s second Coefficient of skewness = 0.07–0.27 for one copy genes compared to 0.01–0.09 for two copy genes; kurtosis = 7.2–10.4 for one copy genes compared to 11.7–13.6 for two copy genes), with thicker tails (two-sided) in the distributions of one copy gene expression values. These data indicate that different genes show differences in compensation responses, continuously ranging from common modest compensation levels through to more rare nearly perfect compensation, with infrequent extreme deviations. We observed similar (but not identical, as will be important later) compensation in females and males (**Fig 1F and 1G**), despite the highly dissimilar gene expression profiles between the sexes ([35], Materials and Methods). Thus, at least some of the response to copy number is a characteristic of an individual gene.

**Fig 2:**
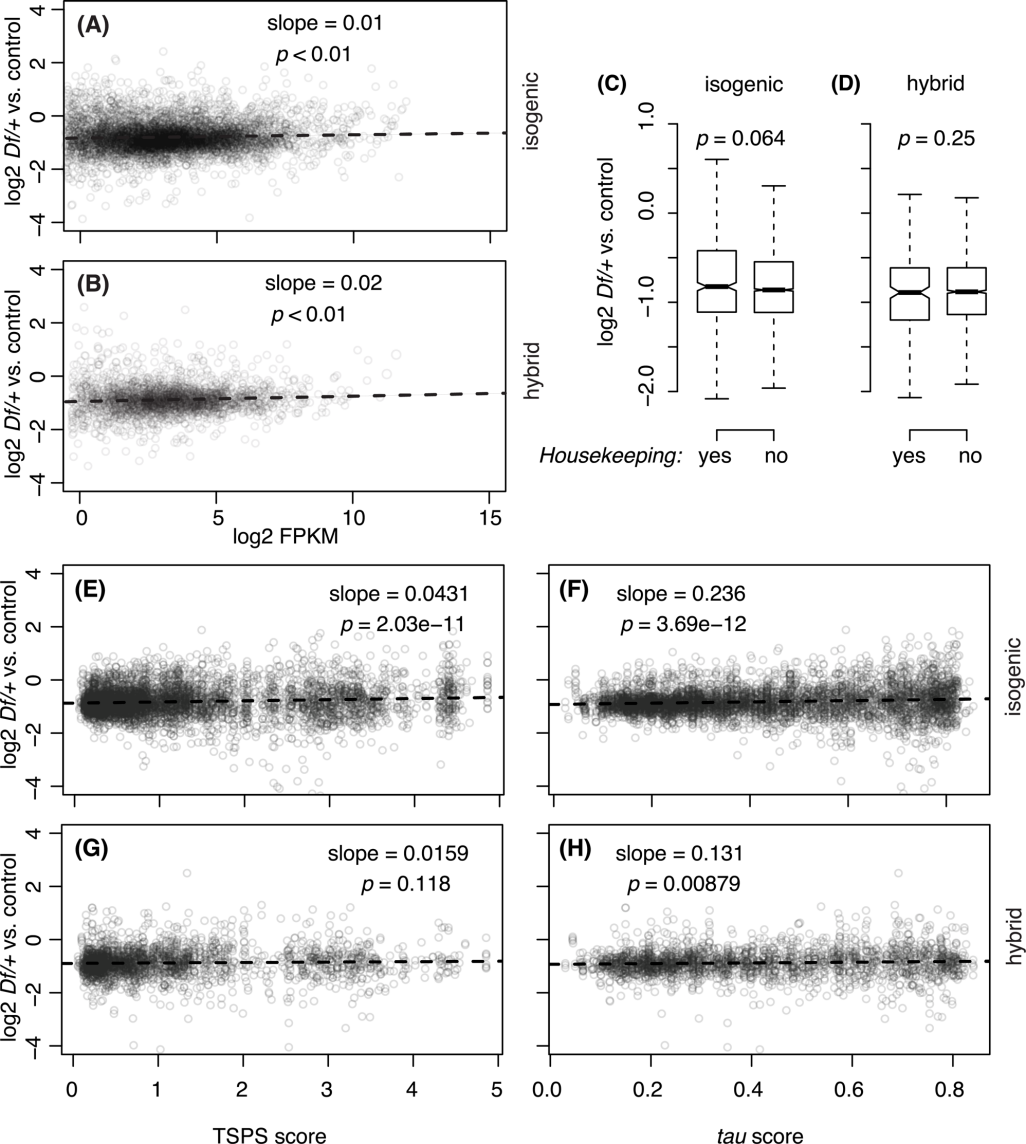
Autosomal dosage compensation of *Df*/+ genes with different expression levels or tissue specificities. A, B) One copy versus two copy gene expression levels plotted against the median expression levels of replicates in Fragments Per Kilobase of transcript per Million mapped reads (FPKM). C, D) Boxplots that display different degree of autosomal dosage compensation of the genes that are classified as housekeeping, or non-housekeeping based on the Naïve Bayes Classifier [40]. *p* values are from Mann-Whitney U tests. E-H) Gene expression levels of *Df*/+ genes were plotted based on Tissue Specificity Score (TSPS, E,G) and *tau* score (F,H). For both scores, larger values indicate more tissue specific. Dashed lines in scatter plots, their slopes and *p* values are from linear modeling and the F-test. A, C, E, F) Results from the isogenic genetic background. B, D, G, H) from hybrid genetic background.

We were interested in determining if there were particular classes of genes or gene functions that might be subjected to different levels of dosage compensation. In particular, increased compensation among genes showing low expression has been noted in several previous studies in *Drosophila melanogaster* [8, 13], which is unsurprising since genes with low expression are expected to be more sensitive to noise and therefore might require tighter expression level control. However, we observed no increased compensation for genes expressed at low levels in our study. In fact we observed marginally better compensation at high gene expression levels (**Fig 2A and 2B)**. The distribution was continuous and independent of low-expression cut-off, indicating that RNA-Seq data handling was not a factor in this disagreement. Low gene expression in whole animals can be due to low uniform expression in most cells or high expression in limited cell types. Compensation has also been reported to be biased for broadly expressed genes in one study [13], although this was not observed in another [8]. We therefore asked if broad temporal or spatial expression heterogeneity correlated with increased compensation using both modENCODE [36] and FlyAtlas [37] expression data as well as three different measures of tissue specificity ("Tissue Specificity score", TSPS [38], "*tau*" [39], and “Naïve Bayes Classifier”, NB [40]). We observed either no significant trend (NB, *p*>0.05), or a modest but significant decrease in compensation among genes with broad expression patterns in these tests (**Fig 2C–2H**). Thus, our data does not support the idea that broadly expressed genes are better compensated. We also asked if Gene Ontology (GO) terms correlated with compensation. When we looked for GO term enrichment in the top 5% of compensated genes using GOrilla [41], we observed only a single enriched GO term (*p* < 0.001), which was the very broad parent "regulation of biological process" in isogenic males, but there was no GO term enrichment related to compensation levels in hybrid males or in either of the female sample types. Thus, while the dose response has a gene-specific component, we were not able to explain this response by particular gene expression levels, expression specificity, or functional gene categories. Compensation is a property of genes with diverse functions.

### Gene-specific dosage response examples

**Fig 3:**
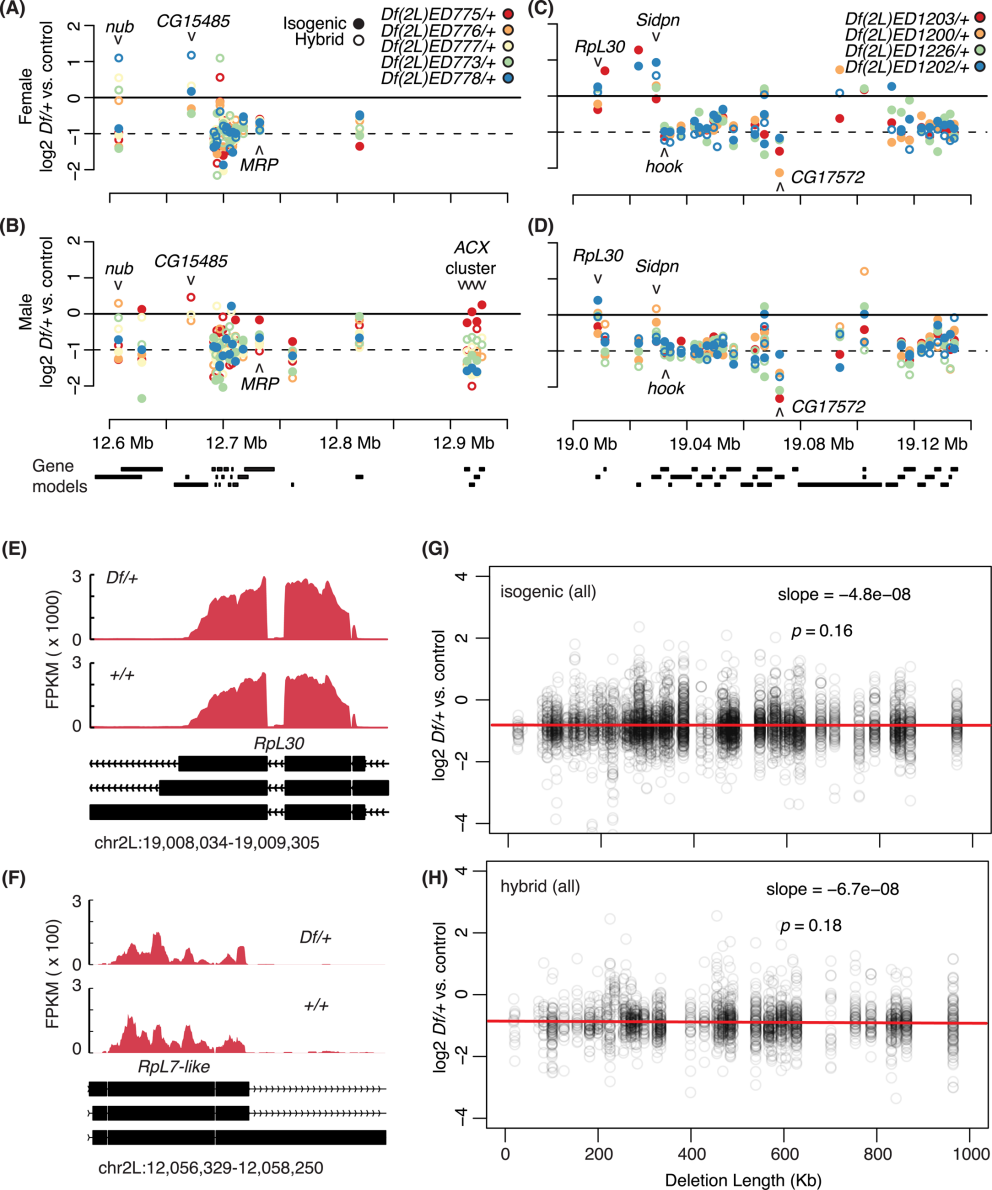
One copy gene responses in different *Df/+* settings. A-D) One copy versus two copy gene expression levels plotted at the centers of each gene model (labeled below). Observations made in the isogenic genetic background (filled) and the hybrid genetic background (open) are shown. Genes whose expression is below the expression cutoff (see Methods) are not shown. A, B) One copy gene expression of the genes between chr2L:12,545,800 and 12,975,028, which is uncovered by five different *Dfs*. C, D) One copy gene expression of the genes between chr2L:19,003,398 and 19,158,447 region that is uncovered by four different *Dfs*. E, F) Sashimi plots that display normalized numbers of mapped reads across *RpL30* and *RpL7-like* gene body regions. Expression in *Df(2L)ED1202/+* for *RpL30* and *Df(2L)ED761/+* for *RpL7-like* was compared to expression in *Df(2L)ED774/+*, which was the shortest deletion in our study and is *+/+* for both genes. Exons (black bars) in the gene models and transcription direction (chevrons) are show below. G, H) Dosage responses (y-axis) of one copy genes when uncovered by deletions of indicated length (x-axis). Results from the isogenic genetic background (top) and from the hybrid genetic background (bottom) are shown.

To further explore the influence of locus, sex and genetic background on the dosage response, we used overlapping *Dfs*. This analysis has the added advantage of determining if a particular *Df* used to uncover a gene altered the response. For example, we examined a region near the middle of *2L* (cytological regions 33–34) with five distinct *Dfs*, and a second closer to the centromere (cytological regions 36–37) with four different *Dfs* (**Fig 3A–3D**). We observed a complex variety of expression variance patterns, compensation responses, and sex- or allele-biased compensation depending on the individual locus. For example, we observed a wide ranges of responses to reducing the dose of the *nubbin* gene (*nub*), from over-compensated (>2-fold increase) to anti-compensation (>2-fold decrease). Differences in dose responses between lines, or even within line, given the possibility of uncovering rare recessive alleles even in highly inbred flies, could be due to differences in cis-regulatory regions in the single copy genes or due to network interactions. The *nub* locus showed some allele-specificity, as we observed better compensation from the *modENCODE OregonR* allele (**Fig 3A and 3B**). In contrast the *hook* gene showed no compensation across 24 different experiments (**Fig 3C and 3D**). We observed partial compensation of the *Multidrug-Resistance like Protein 1* (*MRP*) locus in females (**Fig 3A**), but variable compensation in males (**Fig 3B**). We also observed a sex-biased response in the case of *Similar to deadpan* (*Sidpn*), which was over-compensated in females (**Fig 3C**) and partially compensated in males (**Fig 3D**). At least for these examples, the sex differences are likely to be due to differences in gene network interactions because identical one copy alleles, as well as their regulatory elements, are exposed by the deletions. As expected, based on the correlation between compensation in females and males across *2L* (see **Fig 1F and 1G**), we found that many genes showed similar responses in the sexes. For example, we observed over-compensation of *CG15485* (**Fig 3A and 3B**) and anti-compensation of *CG17572* (**Fig 3C and 3D**) in both sexes. We note with interest that the ribosomal-protein-encoding *RpL30* locus (**Fig 3C–3E**) showed evidence of compensation, consistent with the very weak Minute phenotype reported for mutations in this gene [6]. A second ribosomal-protein-encoding gene (*RpL7-like*, **Fig 3F**) also showed compensation. These two loci are not haplo-insufficient genetically and exceptionally well compensated at the transcript level, supporting the idea that stoichiometric mRNA levels of genes encoding ribosomal proteins are ultimately important for ribosome function [6]. We observed one case where the particular uncovering *Df* correlated with a specific response. In males, the cluster of the *ACXA*, *ACXB*, *ACXC*, and *ACXE* genes (which show male-biased expression) showed very good compensation when uncovered by *Df(2L)ED775*, but much poorer compensation when uncovered by four other *Dfs* (**Fig 3A and 3B**). More generally, in each case the entire ACX cluster of genes correlated better within a *Df* line than between lines. The increased compensation when these genes were uncovered by *Df(2L)ED775* was also allele-specific as the effect was only observed in the isogenic background. The amount of the genome removed by *Df(2L)ED775* was more extensive than most of the *Dfs* used in the study (**Fig 1A**), raising the possibility that the extent of a deletion contributes to compensation. However, we observed no significant relationship between the length of deleted segments and dose responses in our experiments (**Fig 3G and 3H**), suggesting that the extent of a particular *Df* uncovering a locus has little impact on the genes dose response.

### Nuclear architecture and dosage responses

**Fig 4:**
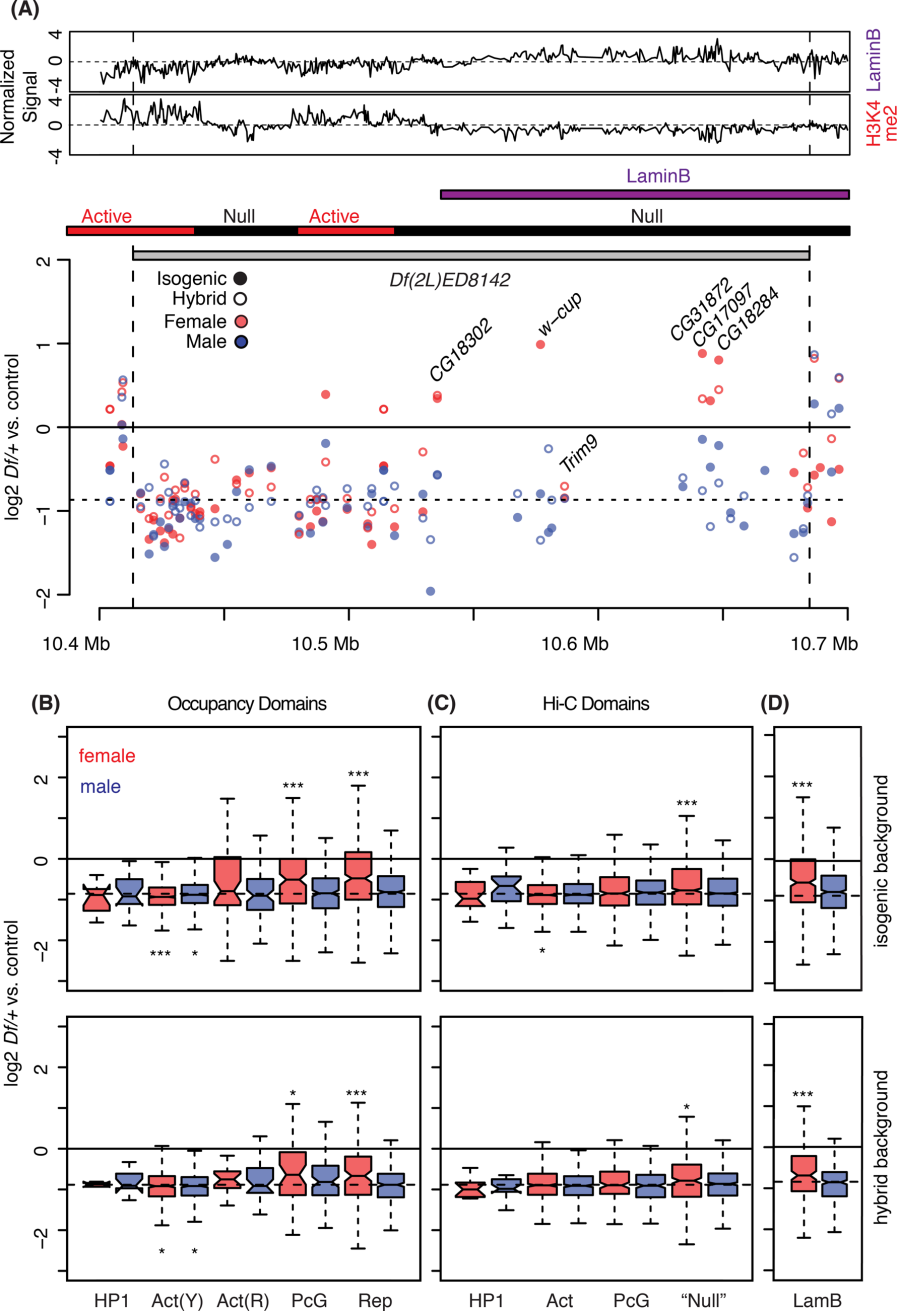
Disruption of chromatin and/or 3D nuclear domains by *Df* breaks. A) One copy gene expression in *Df(2L)ED8142/+* plotted across the deletion position in log2 scale. On the top, DamID (LaminB) or Chromatin immunoprecipitation (Histone H3K4me2) signals (Z-transformed, [42]) across the deletion region are displayed to represent “Active”, or “Null/Repressive” regions, respectively. Chromatin states defined in the Hi-C study [43] and position of LADs from the DamID analysis [44] are presented below the tracks (bars). See Fig 2 for additional labeling information. B-D) Autosomal dosage compensation levels were measured from one copy genes that mapped to different chromatin state domains (B), topologically associated domains (C), and Lamin associated domains (D) from DamID-chip and Hi-C studies. Top panels. Data from the isogenic genetic background (top) and hybrid genetic background (bottom) are shown. Domains are labeled according to diagnostic enrichments/functions from the original studies: Heterochromatin Protein 1 domain (HP1), "yellow" [Act(Y)] and "red" [Act(R)] active domains, Polycomb Group domain (PcG), repressive domain (Rep), undefined and other (Null), and Lamin B (or LAD) domain (LamB). * *p* < 0.05, ** *p* < 0.01, *** *p* < 0.001 (Mann-Whitney U test).

Our data failed to support the idea that specific functional classes of genes or gene features, such as length, expression breadth or level are associated with distinct dosage responses. However, we did notice that some blocks of genes showed common compensation responses and speculated that these might correspond to a particular chromatin state. For example, a group of genes [*CG18302*, *world cup* (*w-cup*), *CG31872*, *CG18284*, and *CG17097*, but not *Tripartite motif containing 9* (*Trim9*)] uncovered by the proximal portion of *Df(2L)ED8142* showed over-compensation in females but not males, while the rest of the genes uncovered by this *Df* showed a more typical partial compensation response (**Fig 4A**). To explore the role of chromatin domains on the dosage response, we plotted our results along with the chromatin structure maps from a DamID (DNA adenine methyltransferase identification) study on chromatin-associated protein occupancy ([42], **Fig 4B**), 3-D structure determination from Hi-C chromatin conformation capture mapping ([43], **Fig 4C**), and nuclear envelope attachment from a LaminB DamID study ([44], **Fig 4D**). In both the genetic backgrounds examined we observed dramatically improved dosage compensation in regions of the genome in structural domains associated with repressed gene expression (**Fig 4B and 4C**). These repressive domains show overlapping characteristics between the DamID and Hi-C studies, and are also enriched in LaminB binding. When we specifically looked at Lamina-associated domains (LADs), we observed improved compensation (**Fig 4D**). We also observed improved compensation in regions of Polycomb group (PcG) protein occupancy (**Fig 4B**), but not in the structural domains enriched in those proteins from Hi-C (**Fig 4C**). Importantly all these improved compensation distributions were observed in females. We found no significant correlations, or even a trend, between repressive chromatin and compensation in males.

In addition to the increased median (and mean) compensation levels among genes within these repressive chromatin domains, we observed an increased range of responses (**Fig 4B–4D**). This suggests that there is greater heterogeneity in the compensation response within repressive domains. We observed modest, but significant decreased compensation in one of the two types of active chromatin in both sexes based on occupancy (**Fig 4B**). Active regions of the "Yellow" type, which is enriched in H3K36me3 domains and in genes with broad expression patterns [42], showed significantly worse compensation, while active regions of the "Red" type showed the same dosage response that we observed globally. These data suggest that in addition to gene-specific responses, the local chromatin state plays a role in dosage responses.

**Fig 5:**
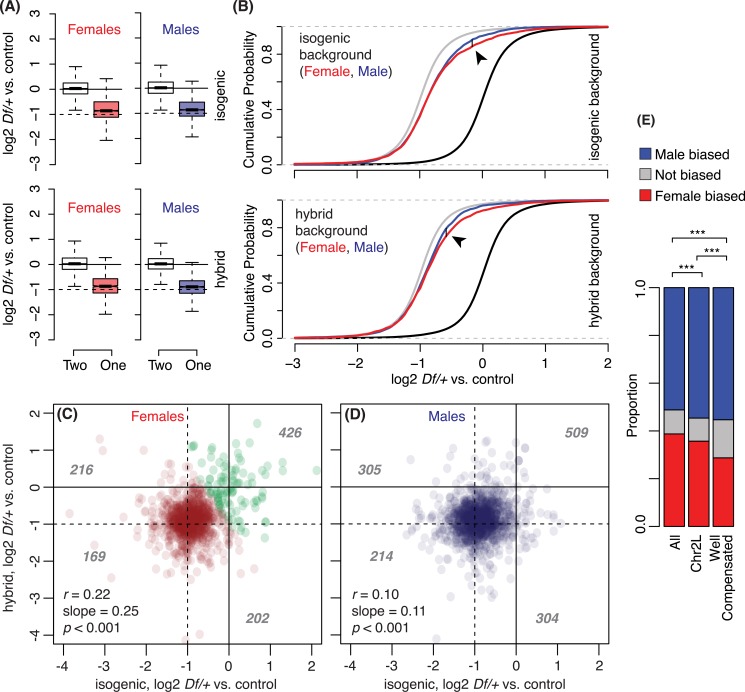
Sex-based difference in one copy gene expression. A) Boxplots of gene expression in two copy genes (open) or one copy genes (red and blue for females and males, respectively) from all *Df* lines used (see Fig 1 for boxplot parameters). B) Cumulative distribution function plots for female *Df*/+ (red), male *Df*/+ (blue), and +/+ gene expression (black, for both sexes). The grey line displays +/+ gene expression level shifted by -1 (log2). Arrowhead indicates Kolmogorov-Smirnov statistic (*D*), the largest vertical differences between two cumulative distributions (0.045 for isogenic and 0.051 for hybrid). A, B) Data from isogenic (top) and hybrid (bottom) genetic backgrounds are shown. C, D) Scatter plots that compare one copy gene expression relative to two copy gene expression between the isogenic genetic background and hybrid genetic background. A subset of genes in (C) represents “better compensated” genes identified in clustering analysis (Green). The grey numbers on the plots indicate the number of *Df*/+ genes appeared in each quadrant, divided by (-1, -1). The same genes that are deficient in multiple Df/+ flies were counted multiple times. Pearson’s correlation coefficient (*r*), slopes from linear regression and *p* values (F-tests) are shown. E) Barplots that display the proportion of male-biased or female-biased genes from all *Drosophila* genes, chromosome 2L genes, and the better compensation in females (C, green). ***p < 0.001 (Hypergeometric test).

Due to the unexpected female-biased compensation of genes within repressive chromatin domains, we examined global sex-biased compensation to see if these blocks of improved compensation are revealed *in toto* and to see if we could detect similar blocks in males. Even if the chromatin states in males differ from those in females, we would expect that weakening repressive domains in males might also result in superior dosage compensation. While median and mean compensation levels were similar in females and males (**Fig 5A**), we observed more genes with very good compensation in females and more genes with intermediate compensation in males (*p* <0.05, Kolmogorov-Smirnov test; **Fig 5B**). When we asked if genes showed similar compensation between isogenic and hybrid backgrounds using expectation-maximization clustering, we observed clear sets of 94 genes with modest and full compensation in females. If repressive domains are in different regions in males, those regions should be identified as better compensating, but we were unable to detect two clusters in males (**Fig 5C and 5D**). These data suggest that there are subtle differences in autosomal compensation between the sexes, as suggested by the correlation between repressive domains and female-biased compensation. At least some of the genes showing compensation in females due to de-repression were highly expressed in male-specific organs. When we asked if the 94 genes consistently compensated in females showed a difference in expression between females and males, we found that genes with male-biased expression were significantly over-represented, and genes with female-biased were under-represented, relative to the genome as a whole or chromosome 2L, which is also enriched for genes with male-biased expression (**Fig 5E**).

The increased compensation when repressive domains are deleted is consistent with a role for chromosome pairing and reinforcing repressive effects that are relaxed by deletion from one homolog. It is important to note that removing the 94 genes highly compensated in females from the analysis has a minimum and non-significant effect on the overall pattern of modest compensation, which remained at 1.1-fold in females. Thus, at the level of a *2L*-wide overview, our comparison between the sexes suggests at least two independent mechanisms for compensation. One is characterized by a varied gene-by-gene response to copy reduction, consistent with a feedback/buffering model for dosage responses (and allele/condition specificity between backgrounds) that accounts for the bulk of the observed compensation. The other is due to chromatin structure in females restricted to a small set of genes, possibly related to repressing male gene expression in females.

### Breakpoints and gene expression

**Fig 6:**
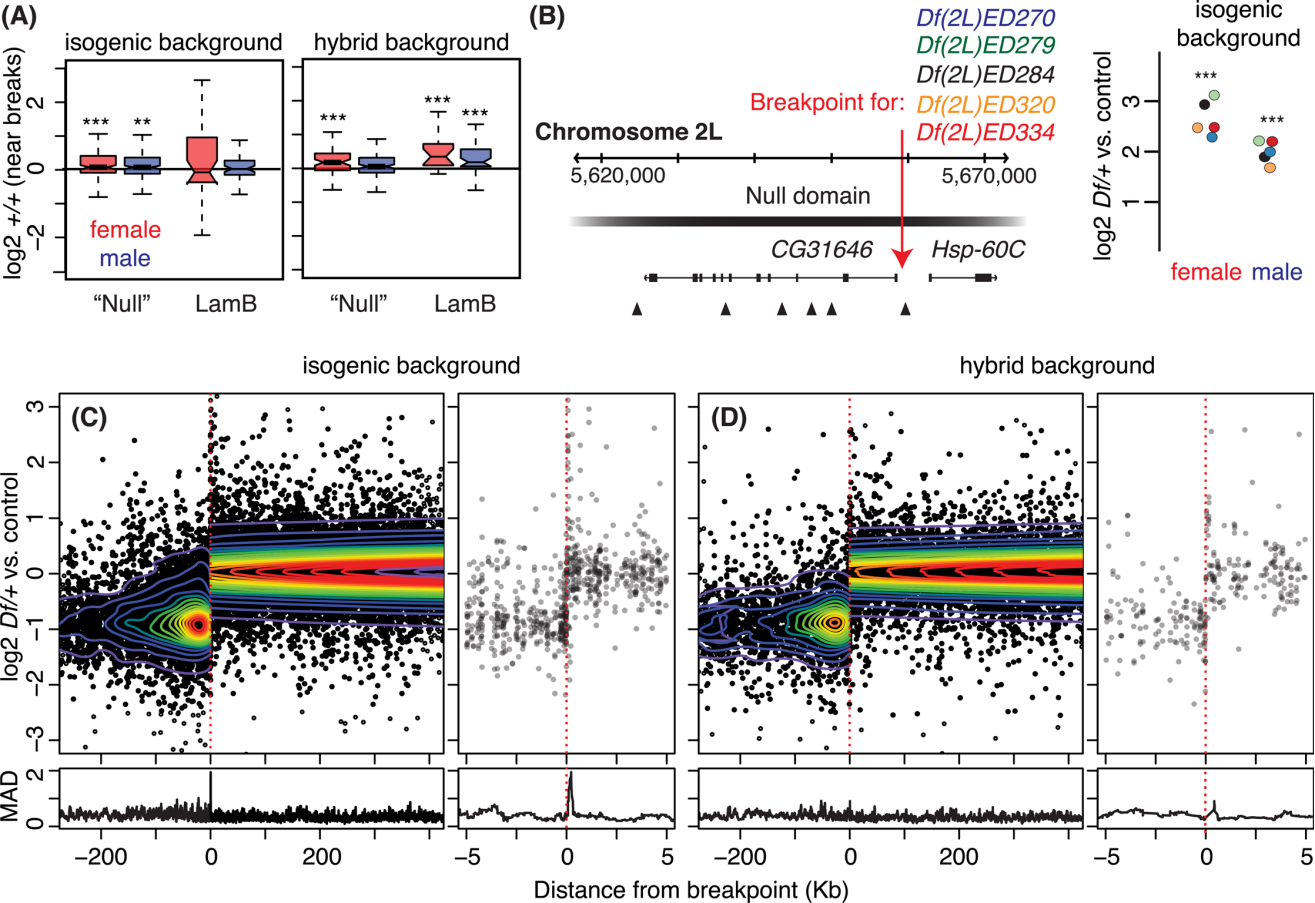
Disruption of DNA linear structure by *Df* breaks. A) Two copy gene expression near *Df* breakpoints. The boxplots display distributions of normalized relative two copy gene expression within the Null domain (from Hi-C), or LADs that are disrupted by deletions (see Fig 1 for boxplot parameters and Fig 3 for statistics). B) A schematic of the CG31646 gene model (left) showing the position of a common breakpoint (red arrow) for the 5 *Dfs*. Chromatin state (Null domain, [43]) is represented as a bar. Triangles indicate insulator positions [46]. Deletions exist on the right side of the common breakpoint. Expression changes of *CG31646* in different *Df* lines are indicated (right). Colors of the filled circles match with the *Dfs* lines by the plot. *p* values (asterisk) are based on empirical Bayes moderated T-test in the limma package (See Methods, *** *p* < 0.001). C, D) Gene expression changes near breaks are collectively displayed by aligning all breakpoints in the study at "0" (red dotted line). One copy genes are placed on the left side, and two copy genes are place on the right side of 0. Contours represent data point density. Wide (left) and zoomed (right) views of the same data are shown. Variability in gene expression was summarized using Median Absolute Deviation (MAD, a non-parametric measure of the variability) from sliding windows of 30 genes (bottom).

Deletions bring distant regions of the chromosome into linear juxtaposition: if such novel genome arrangements fuse domains or destroy insulator elements then they may create new expression environments (**Fig 6A**). If repressive domains can be weakened by deletions *in trans*, as suggested by our data in females, those effects might also spread into adjacent regions of repressive chromatin that are juxtaposed with other types of chromatin *in cis*. This is essentially the opposite of position effect variegation, where spreading of repressive chromatin is observed [45]. Our data may suggest that active chromatin spreads into repressive domains. We observed modest but significant effects on genes flanking breakpoints when the breakpoint was within a previously identified LaminB repressive domain (**Fig 6A**). We found slight and occasionally significant increased expression of two copy genes flanking breakpoints in "Null" Hi-C domains (**Fig 6A**). However, the significance of this effect of breakpoints was variable by both sex and background, tempering the conclusion that ectopic juxtaposition alters gene expression within broken chromatin domains. The other chromatin domains showed no significant correlation with homozygous expression. Extensive studies of position effect variegation in *Drosophila* have clearly established that repressive chromatin can spread into active chromatin when it is brought into that environment by transposition or inversion. Our work supports the idea that spreading also works to weaken the repressive domain [30]. Our data indicates that LaminB repressive domains are sensitive to de-repression, *in cis* or *trans*, by bulk deletion, although the *trans* effect is much clearer. This suggests that LaminB domain repression is additive or cooperative.

Breakpoints also have the potential to create novel enhancer/silencer/promoter interactions. We found limited evidence that removal of cis-regulatory regions by *Dfs* could influence the expression of genes with gene bodies in adjacent non-deleted segments. For example *Df(2L)ED270*, *Df(2L)ED279*, *Df(2L)ED284*, *Df(2L)ED320*, and *Df(2L)ED334* all have a common breakpoint just upstream of the *CG31646* promoter, deleting a region where enhancers and silencers are often located, and in this case deleting a known CNS regulatory region [47] (**Fig 6B**). In males, and especially in females, these deletions resulted in dramatic over-expression of *CG31646* suggesting that a silencer was removed by each of these deletions. To determine how common the effects of structural rearrangements on gene expression are, we centered all the breakpoints from the *Dfs* used in this study and plotted expression flanking the breakpoint as well as the median absolute deviation to summarize the results. We observed no significant change in expression with distance from the breakpoint, with the exception of genes within 100bp of a breakpoint (**Fig 6C**). Even this breakpoint proximal effect is probably less significant than it appears, since the spike of increased expression in the isogenic background (**Fig 6C**) is due almost exclusively to the *Dfs* in **Fig 6B**. Thus despite the fact that the deletions in our study removed a total of 2,100 insulator binding regions identified in an embryo study [46], and break within 437 chromatin domains from the Hi-C study [43], we find little evidence that this plays a major role in transcription.

### Propagation through gene networks

The general absence of breakpoint proximal effects of *Dfs* on transcription of two copy genes does not mean that there is no effect of deletions on the rest of the genome. We observed tens of significant changes in gene expression for each gene made single copy in a *Df/+* fly (**S1 and S2 Files**). In total, we observed 10,757 two copy genes that displayed significant (adjusted *p* < 0.05) changes in expression in at least one *Df/+* line. Overall, 78% of genome changed expression in this study as a result of altering the gene dose of 15% of the genome.

**Fig 7:**
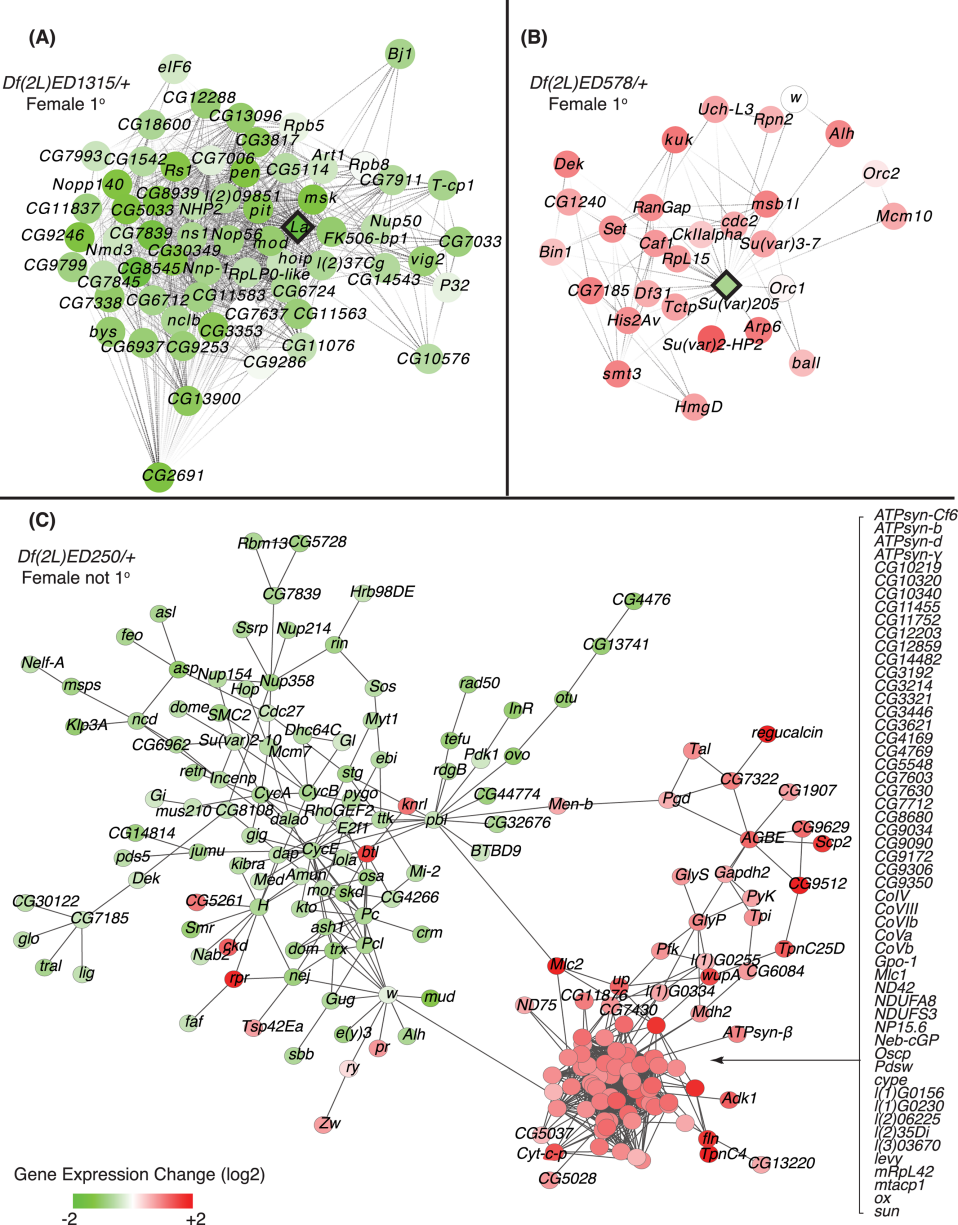
Propagation of gene dose perturbation in gene networks. Nodes represent genes and their connections, or edges, show interactions defined in the integrative network model. A, B) Subnetworks that include *Df* genes (diamond nodes) and their 1° two copy neighbors (round nodes) in the gene network. Gene expression changes in *Df(2L)ED1315/+* and *Df(2L)ED578/+* females have been projected onto the network. Up-regulated (red) down-regulated (green) gene expression relative to controls, as well as no change (open), is indicated with shading showing the magnitude of expression change (see key at bottom). C) Identified functional module that is significantly differentially expressed in *Df(2L)ED250/+* females, but with an unknown connection to a one copy gene(s).

To determine if two copy genes that changed expression fell into clusters of genes related by network interactions or were more randomly organized, we projected gene expression changes onto an integrated network model (non-directional), based on expression in published GEO datasets, gene interactions, and protein-protein interactions, which is largely consisted with positive interactions [48]. We observed striking examples of propagating effects in network space. In the movie (**S3 File),** we show the propagating expression change as we tiled through chromosome 2L. Coherent changes in gene expression occur in specific sub-regions of the network, and these differ between females and males. In the majority of cases, there was a positive correlation between gene expression in the one copy gene and in the two copy network neighbors. For example, hemizygous *La autoantigen-like* (*La*) expression was reduced in *Df(2L)ED1315/+* females, as were a number of other genes that are primary (1°) network neighbors of *La* (**Fig 7A**). These genes show enriched expression in ovaries, larval central nervous system, and larval trachea, but are down-regulated in other tissues [37], and are highly enriched in genes encoding ribosome biosynthetic machinery according to GO term analysis (*p* << 0.001, Holm-Bonferroni corrected Hypergeometric test). This positive relationship between hemizygous expression and expression in network neighbors was more prevalent, but propagation patterns also showed negative interactions. As an example of a negative interaction, is the cluster centered on the *Suppressor of variegation 205* (*Su(var)205*), which encodes heterochromatin protein 1 (HP1) that binds H3K9me2/3 and is a general repressor of transcription [49]. Hemizygosity for *Su(var)205* in *Df(2L)ED578/+* females resulted in reduced expression of this negative regulator. The primary neighboring genes connected to *Su(var)205* in the network model all showed increased expression (**Fig 7B**), consistent with de-repression when HP1 levels are reduced. Globally, there is a nearly equal number of two copy genes that show elevated or reduced expression in response to a *Df/*+ condition. These changes occur at a greater distance from the one copy gene source. For example, we observed coherent changes in pathways (statistically significant network module clusters [50]) that we could not directly connect to one copy genes. These were often connected with a few edges to large groups of genes showing the opposite effect. For example, in *Df(2L)ED250/+* females, a strong cluster of genes with ovary-biased expression in wild-type (89% have transcripts enriched in ovary [51]) are down-regulated (**Fig 7C**). These include *Cyclin* genes, which are likely expressed in the replicating germline and somatic support cells of the ovary (*CycA*, *CycB*, and *CycE* [52, 53]), the important female germline transcription factor *ovo* and the known Ovo target gene *ovarian tumor* (*otu*) [54, 55]. Down-regulated genes in this ovary-related group also include many targets of the somatically expressed Doublesex transcription factor, such as *Grunge* (*Gug*), *domino* (*dom*), and the *Insulin receptor* (*InR*) gene [56]. This cluster of genes significantly enriched for functions in oogenesis is linked to an even larger cluster of up-regulated genes, 77% of which encode components required for oxidative phosphorylation. Energy storage molecules are deposited in the egg to support embryonic development and are converted to ATP if not stored. We suggest that our observations reflect physiological pathway compensation in *Df(2L)ED250/+* females, where down-regulation of oogenesis results in increased catabolism. In terms of propagating effects of one copy genes, the effects on two copy genes with functional rather than physical proximity to one copy genes strongly indicates that expression changes in the diploid portion of the genome are due to regulatory pathways, not structural changes in the genome or long range contacts.

**Fig 8:**
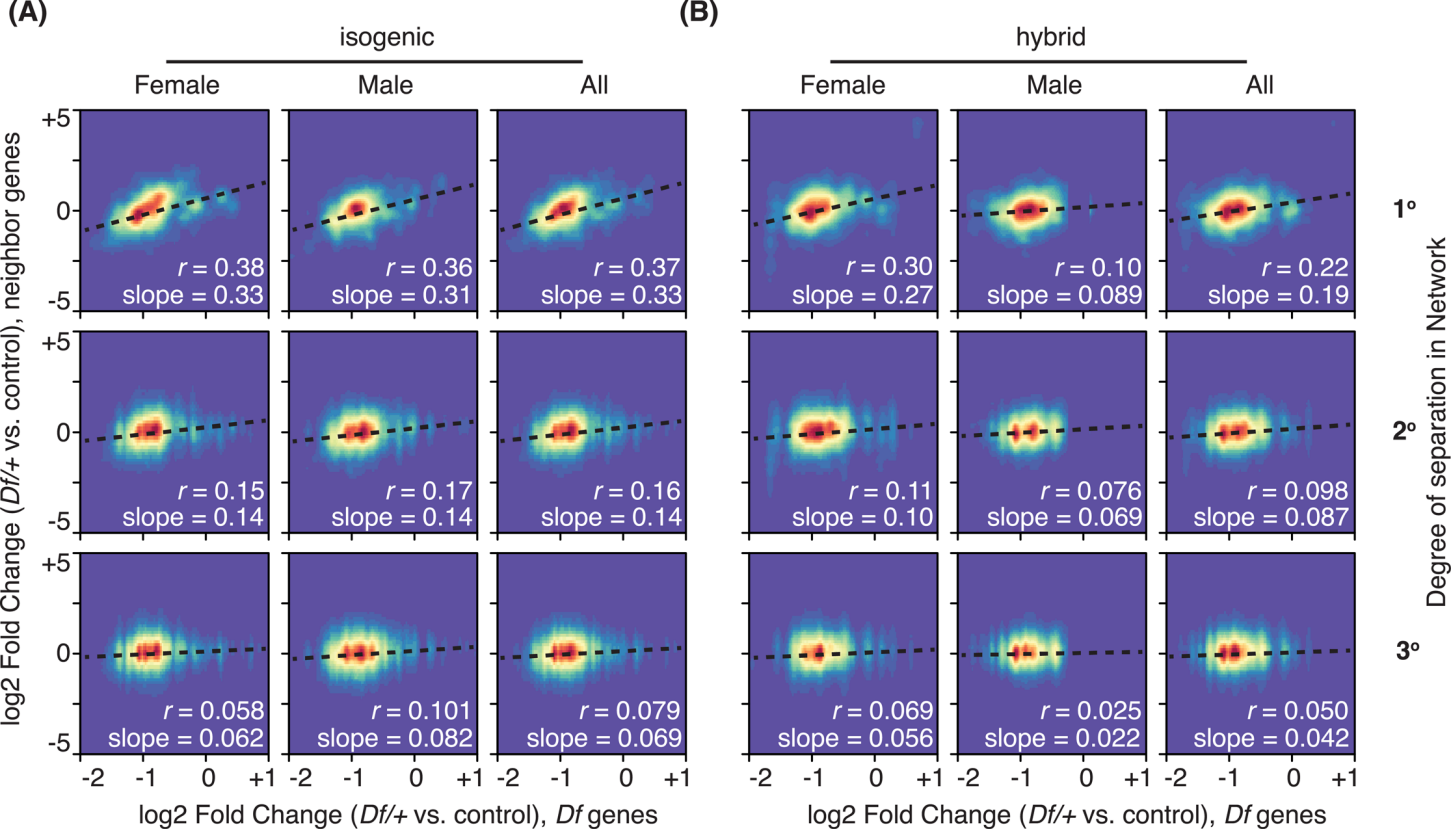
Propagation and dissipation of gene dose perturbation in positively correlating networks. Density plots that display gene expression changes of two copy genes that are neighbors of *Df* genes in the integrative network by 1° (primary, top), 2° (secondary, middle) and 3° (tertiary, bottom) are collectively plotted from all used lines against the expression changes of *Df* genes (one copy, x-axis) in females (left), males (center) and combined (right). Results from both the A) isogenic and B) hybrid background are shown. Pearson’s correlation coefficient (*r*) and slopes from linear modeling are shown.

We examined the global relationship between expression of one copy genes and their two copy network neighbors in the entire date set (**Fig 8**) and observed a strong correlation in the expression of one copy genes and their 1° network neighbors. As network distance increased this correlation degraded and was not distinguishable after 3° connections. This is consistent with a gradual dissipation of relation to the driver perturbation due to the action of corrective feedback responses and passive buffering at each step. These expression pattern relationships were consistent between the sexes and in both the isogenic and hybrid backgrounds.

### Propagation robustness

**Fig 9:**
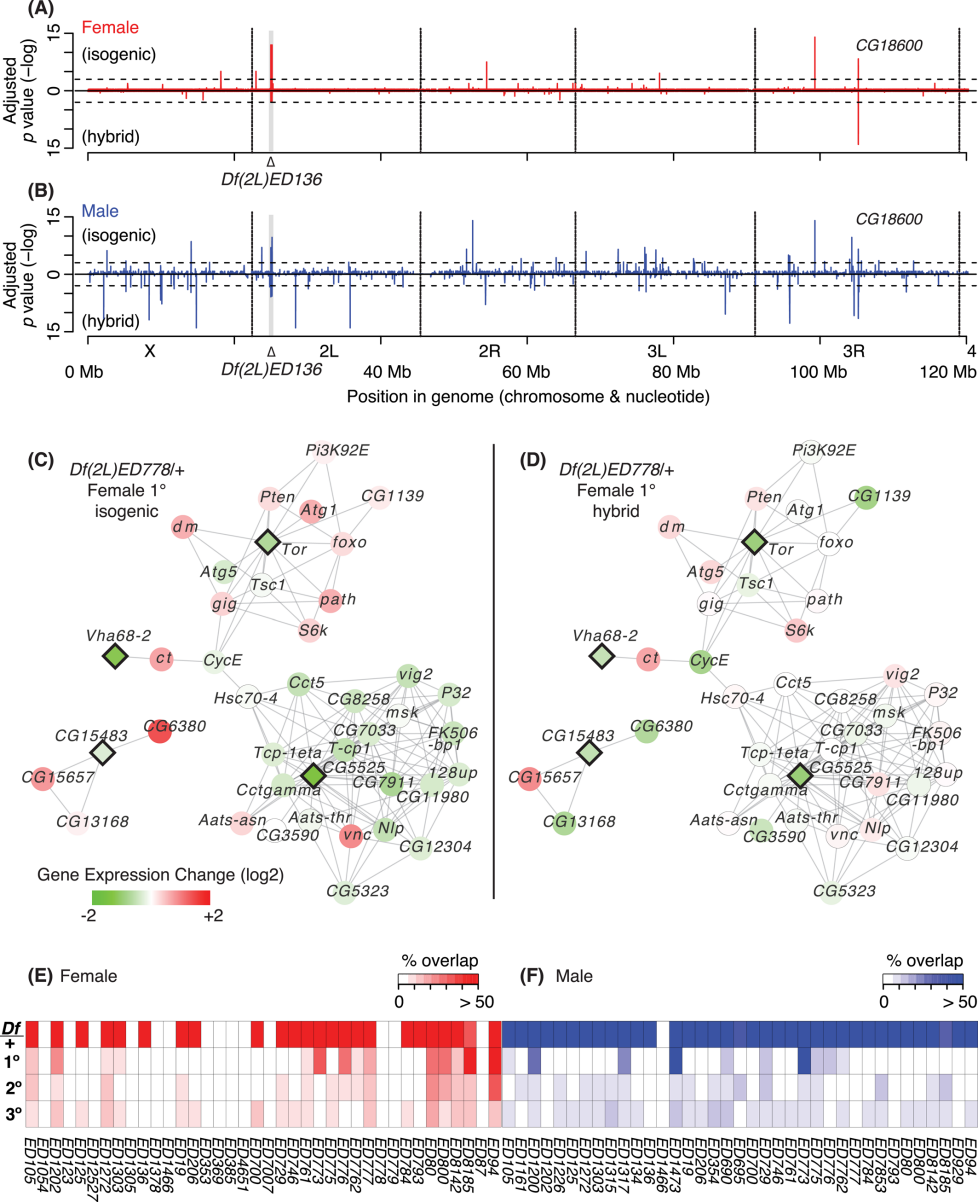
*Df.* causes genome-wide gene expression change in a genetic background-dependent manner. A, B) Adjusted *p* values of gene expression change is plotted in–log scale across the genome for females (A) and males (B) of *Df(2L)ED136/+* flies. The grey region on chromosome 2L indicates the deletion. The threshold adjusted *p* = 0.05 is shown (dotted lines). *CG18600* displayed significant changes in both genetic backgrounds and sexes. C, D) Gene expression changes of *Df*/+ genes (diamond) and their two dose 1° neighbors (circle) genes in the integrated gene network of female *Df(2L)ED778*/+ flies from isogenic (C) and hybrid (D) genetic backgrounds. E, F) Overlap of gene expression changes between *Df*/+ genes, and their two dose 1°, 2°, and 3° neighbors between two different genetic backgrounds in females (E) and males (F). We called genes whose changes are greater than 1.5 folds and show same directional changes (up or down) in both genetic backgrounds to be overlapped. For brevity, “*Df(2L)*” and “*/+*” are omitted in genotypes. E.g. *Df(2L)ED105/+* is shown as *ED105*.

We observed similar overall patterns of network propagation and dissipation in both the isogenic and hybrid backgrounds in both sexes. However, the precise genes that changed in response to a given *Df* differed by sex and by genetic background. For example, *Df(2L)ED136/+* males showed many more expression changes than in females in both backgrounds (**Fig 9A and 9B**). In males, other than the one copy genes, only four genes showed a significant expression change in both backgrounds. Of the genes showing differential expression in *Df(2L)ED136/+* males, only *CG18600* was also differentially expressed in females. This gene is expressed preferentially in the ovary, as well as in the testis and male accessory gland [57]. Our data suggests that there is common regulation of *CG18600* in these different organs, mediated by a gene deleted in *Df(2L)ED136*. Given the pervasive sex-bias in *Drosophila* gene expression, we expected to see different network responses between females and males, but the extent of this dichotomy was striking.

The context-dependent propagation of expression change was a marked common trend, as illustrated by the example of *Df(2L)ED778/+* females (**Fig 9C and 9D**). These flies have one dose each (clockwise from the top) of *Target of rapamysin* (*Tor*), *CG5525*, *CG15483*, and *Vacuolar H*^*+*^ *ATPase 68 kDa subunit 2* (*Vha68-2*). The network neighbors of these genes change in both backgrounds, and these are sometimes the same. For example, the gene pairs: *Tor* and *Ribosomal protein S6 kinase* (*S6k); CG15483* and *CG15657*; and *Vha68-2* and *cut* (*ct*) show the same responses in both backgrounds. This is not always the case, as there are discordant gene pairs such as *Tor* and *CG11389*, and *CG15483* and *CG6380*.

These results can make clear predictions for subsequent experiments. For example, the genes surrounding *Tor* function in insulin and autophagy related pathways, with activated TOR suppressing autophagy in *Drosophila* [58]. In the isogenic background, reducing *Tor* dose correlates with higher *Autophagy-related gene 1* (*Atg1*) expression, whose over-expression is a strong driver of autophagy [59]. Interestingly, *pathetic* (*path*) is up-regulated in the same genetic background. *Path* encodes amino-acid transporters that function as nutrient sensors [60] and it is possible that local sensing of amino acids activates S6K proteins to compensate for low TOR levels. There is a large cluster of genes surrounding *CG5525* [61], which also show an isogenic background specific response. These genes are enriched for molecular chaperone functions, including those encoding members of the Tailless-Complex-Polypeptide-1 complex [62]. Dose-dependent down-regulation of *CG5525* is positively correlated with down-regulation of most of the cluster in the isogenic genetic background. Misfolded proteins cause autophagy [63], and there also is a chaperone-dependent mode of autophagy [64]. This example shows how *Df/+* profile exploration is a strong hypothesis generating exercise.

Globally, even though we observed similar rates of propagation through 1°, 2°, and 3° network neighbors, the same neighbors were rarely implicated in the different genetic backgrounds (**Fig 9E and 9F**). Thus, while there are coherent pathway responses to hemizygous driver perturbations, the exact path through network space was highly dependent on sex and some combination of genetic background and environment.

## Discussion

The large collections of deletions are widely used tools by the *Drosophila* research community, representing the most commonly ordered stocks from the Bloomington Drosophila Stock Center. Unfortunately, we know very little about the effects these deletions have on the global fly transcriptome and here we describe our initial efforts at addressing this issue. We have touched on three aspects of the effects that deletions have on the transcriptome: 1) the primary effect of hemizygosity on gene expression; 2) juxtaposition of regions of the genome that are normally distant; and 3) the effects of deletions at a distance in either network or physical space.

### Dosage effects and compensation

In a deficiency, there is only expression from only one allele of the genes within the deleted region, and while this generally results in expected reductions in gene expression, the response of an individual gene to copy number reduction is highly dependent on feedback regulation [8] and buffering, both of which are inherent properties of biochemical pathways [8, 10, 12, 13]. Different degrees of median autosomal compensation have been reported for *Drosophila melanogaster*, ranging from no compensation to nearly 2-fold up-regulation of one copy gene expression [9–11, 13–15, 19]. Similar results have been observed for X chromosome dosage compensation in *Df/+* females [14], or in males prior to the onset of X chromosome dosage compensation mediated by the MSL complex [65, 66]. Our compensation values in this report are at the low-end of previous estimates. Because the question of whether non-sex chromosome compensation exists and if so to what degree is a generic problem in biology, as shown in recent debates in the yeast community [17, 18, 67], we will outline some important factors that contribute to the different levels of compensation reported.

Some of the differences in compensation values are probably due to biology. For example, individual *Dfs* have distinct compensation levels because of the set of genes uncovered have gene-specific dosage responses. Thus, results will vary depending on exactly which region of the genome is examined. This report and our earlier work [14] have accumulated enough RNA-Seq samples (N = 1062 in GEO, GSE60571, GSE73920, and GSE61509) to indicate that 1.1-fold overall compensation value in whole adult flies is robust to random sampling approaches. It is not clear if genes show the same compensation responses in all tissues and all stages of development, but we observed no difference in compensation between heads [14] and whole bodies (this report). The most extreme example of biological confounding factors is the varied dose responses of aneuploid tissue culture cells [15]. The dose effects for essentially the entire genome have been probed in highly aneuploid *Drosophila* cell lines [15, 16] with the observation that changes in copy number are not random. Multiple independent changes in gene dose are identified in these studies, suggesting that cell lines evolved copy number states. It is also clear that they show variable degrees of dosage compensation, which is also likely to be evolved. For example, *Drosophila* Sg4 cells show nearly complete dosage compensation, while D17-c3 cells show no evidence of compensation [15]. Tissue culture cells show recurrent copy number changes that are likely to enhance culturability, which would be counteracted by compensation. In contrast, it is also possible that better compensating cells have a selective advantage, resulting in higher gene dosage compensation levels in many lines. Finally, it is also possible that some cell lines have evolved other mechanisms to cope with copy number changes. For example in yeast, a mutation in de-ubiquitiniating enzyme *Ubp6* make cells more tolerant of aneuploidy [68]. Highly aneuploid tissue culture cells might be good models for some aneuploid states, such as those occurring in cancers, but selection for growth is powerful and these cells may not be suitable models for understanding generic dose responses.

In at least some cases, we believe that compensation has been over-estimated for technical reasons. Data compression in microarray-based studies contributes to over estimating dosage compensation especially at low expression levels where array responses are nonlinear [69–71]. This occurs despite strong efforts to estimate non-specific background hybridization. If stringent expression cutoffs are applied to array data used to measure compensation levels [10], the observed average compensation is approximately1.1-fold, the value we report here. Reanalysis of array data showing 1.4-fold compensation [8] with the same stringent method also results in 1.1-fold compensation. Early sequencing-based studies may have similarly over-estimated compensation. For example, the original RNA-Seq study of *Drosophila* S2 cells reported approximately 1.3 to 1.4-fold compensation [16], however, employing more advanced sequence aligners, copy number callers and improved gene annotation yields a 1.2-fold compensation value from the same raw dataset [15]. Unfortunately, some studies use reported summary values from papers as a baseline for comparing to new results from a different data analysis pipeline and/or discordant biological materials. This should be avoided. Making very small fold change measurements requires tight internal controls.

### Gene-by-gene compensation mechanisms

While dosage responses are often bundled together under terms like buffering or compensation, this does not mean that there is a single mechanism. We find clear examples of genes showing no compensation, indicating that transcript abundance for these genes is uncoupled from the expression changes occurring elsewhere in the genome. This is a surprising observation since transcriptional accumulation is the result of a chain of enzymatic reactions subject to general rules of kinetics, where the accumulation of final product levels is not a simple consequence of the dose of a single component, but rather the flux through the system [27, 72, 73]. Assuming that many of the components of this passive buffering will affect all genes, perhaps monotonically, it seems likely that modest compensation we observed along chromosome 2L is due, at least in part, to this mechanism. The cases of collapsed or over-compensation, are best understood as interruptions of active regulatory circuits.

### Compensation by regional de-repression

There has been debate about whether there is a regional response to reduced gene dose in *Drosophila* [8, 10, 12, 13]. In our analysis of chromosome *2L*, we found that most contiguous groups of genes showed different dosage responses. However, we also identified blocks of well-compensated or over-compensated genes in females. Interestingly, the chromatin domains resulting in superior compensation were repressive, with diagnostic LaminB and/or PcG enrichment. The PcG proteins can mediate pairing-dependent silencing [74], which has the counter-intuitive effect of increasing expression of one copy genes. We observed the same effect in some clusters of one copy genes in this study. Thus, some of the strongest compensation we observed is likely to be due to de-repression *in trans*.

Curiously, we observed this regional LAD and PcG dosage compensation response only in females. *A priori*, there should be no reason that de-repression should be female-specific. For example, we are aware of no reports of PcG paring-dependent silencing being restricted to females. Formally, it is possible that female-specificity is due to ascertainment bias. The small set of genes showing compensation in females was found in a subset of the *Dfs* analyzed. Those genes also tended to show male-biased expression. Chromosome 2L is especially enriched in genes with male-biased expression, so it is possible that genes with female-biased expression are repressed by a similar mechanism in males elsewhere in the genome. Additionally, we used domains defined by work in female tissue culture cells and non-sexed embryos [42–44] and compared these to adults. It is possible that the arrangement of domains in the adult fly could be significantly different and sex-biased. However, we did not observe blocks of high compensation in males regardless of information on chromatin state, making this scenario less likely. It also appears that there are sex-specific differences in the nature of heterochromatin in *Drosophila* [75], although the nature of these differences is not fully understood. Compensation by de-repression could be ovary-specific for example.

### Breakpoints

*Df.* breakpoints bring two regions of the genome together that are usually distant in the linear chromosome. This can result in transcription unit fusion as occurs in many cancers and has been especially well studied in immune cell tumors [76]. We observed only one such case in our analysis. *Df(2L)ED680/+* results in a fusion transcript of *taiman* and mini-*white* (a marker associated with the engineered deletion). Given that the deletions were made from insertions of P-elements, which have a preference for 5’ gene regions and promoter regions [77], the low frequency of genes fusion events may not be typical of all deletions.

It is also clear that some genes have enhancers and silencers located many kilobases from the promoter [78–80]. If these are common cases, then they should often be deleted at promoter proximal breakpoints while leaving the transcription unit intact. Additionally, the genome is organized into chromatin domains flanked by insulator sites, which could facilitate regional transcriptional control by "opening" or "closing" multi-gene segments of the genome [81]. Deficiency breakpoints we used delete thousands of insulator binding sites resulting in the creation of novel arrangements of insulator pairs, which should fuse many chromatin domains. If this creates a novel gene expression regulatory milieu, then transcription should be altered. However, our analysis indicates that, at least across ~ 20Mb of the genome we surveyed, the vast majority of the regulatory information is within the gene body or ~100bp upstream. This agrees with work where inversions were generated within neighborhoods of co-expressed genes, which failed to disrupt co-regulated gene expression in *Drosophila* [31]. However, this is in stark contrast to the studies on genes involved in early pattern formation, which show clear long-range regulatory interactions [82, 83]. This is also in contrast to the assumptions in many studies of 3D nuclear architecture, where long-range interactions that can be mapped, are assumed to be functional [84]. While it is quite clear that a definable nuclear architecture exists, and is likely critical for packaging long DNA molecules into the confines of the nuclear envelope, it is much less clear that this topology is required for gene regulation.

We have no evidence that the absence of long-distance interactions is due to ascertainment bias, but this is possible. The *Df* collection was made using recombination between insertions, but some targeted *Dfs* were not recovered (~25%). It is possible that these non-recovered *Dfs* disrupted long-range interactions resulting in dominant lethality [85]. Even if this is true, our data indicate that the many DNA topological domains and potential long-range enhancer promoter interactions we directly interrupted have minimal effects of transcript abundance. Only the regional effects of repressive domains appear to be an exception and these effects on genes flanking breakpoints were weak. This strongly suggests that much transcriptional regulation is local and is unlikely to be influenced by a particular chromosome domain structure or distant silencers and enhancers.

### Network interactions

We observed substantial changes in gene expression throughout the genome, not just in the hemizygous regions, and these are likely due to "error" propagation as is expected in a dynamic biological system. Propagation appears limited to the first few nodes in the network model, although substantial changes that are hard to link to the initial dose perturbation are also seen. That the primary two copy network neighbors of one copy genes change expression in response to reduced dosage of genes uncovered by deletions is unsurprising, since decades of work in *Drosophila*, has identified many dose-dependent enhancers and suppressors by screening with deletions. Such dominant genetic interactions are very valuable for finding near neighbors in genetic pathways. For example, much of what we learned about the germline sex determination pathway in *Drosophila* began with screens identifying pairs of interacting genes. The *sans fille* (*snf*) and *fl(2)d* loci show dominant interactions with *Sex-lethal* (*Sxl*) resulting in germline tumors [86, 87], and are now known to be components of the splicing machinery participating in *Sxl* autoregulation [88]. Similarly, screening *Df* heterozygotes for dominant interactions with *ovo*^*D*^ identified a number of interacting genes [89], including the direct Ovo target, *ovarian tumor* (*otu*) [55].

Our data suggests that expression profiling *Df/+* flies can identify candidate pathways and interactions with different *Df/+* altering distinct pathways in the adult. In many cases *Dfs* reveal tightly connected subnetworks of genes expressed in a particular tissue, such as the gonads, gut, or eyes. The precise genes that change expression are different between the sexes or genetic backgrounds. This suggests that in screening a *Df/+* kit for interactions control of background is important whether it occurs by crossing in test mutations, or inherent in the kit because of the diversity of backgrounds and labs that have contributed *Df* lines to the stock center over the decades. In addition, it is not necessarily expected that overlapping *Dfs* will show the same dose-dependent modifications, especially if they were generated in different screens, and even within the same background each *Df* results in a distinct propagation pathway. It may therefore be more useful to think of genetic interaction screens as probing pathways. Gene dose perturbation appears promising for understanding complex information flow in gene networks that may contribute to building better network models. This large solution space for network information flow poses a great challenge to our understanding of complex genotypes. Predictive models will be required.

## Materials and Methods

### Fly lines

*Drosophila melanogaster* were raised at 25°C on standard yeast/cornmeal medium (Fly Facility, University of Cambridge, UK) for *w*^*1118*^ isogenic flies, and on standard fly agar (Bloomington Drosophila Stock Center, Indiana University, IN) for *w*^*1118*^/*OregonR* hybrid flies. Virgin *w*^*1118*^ or modENCODE *OregonR* females were crossed to DrosDel strain males and the non-balancer flies were collected and aged for 3–5 days.

We used the following DrosDel lines: *Df(2L)ED105*, *Df(2L)ED1050*, *Df(2L)ED1054*, *Df(2L)ED108*, *Df(2L)ED1109*, *Df(2L)ED1161*, *Df(2L)ED1196*, *Df(2L)ED1200*, *Df(2L)ED1202*, *Df(2L)ED1203*, *Df(2L)ED1226*, *Df(2L)ED123*, *Df(2L)ED125*, *Df(2L)ED12527*, *Df(2L)ED1272*, *Df(2L)ED1303*, *Df(2L)ED1305*, *Df(2L)ED1315*, *Df(2L)ED1317*, *Df(2L)ED134*, *Df(2L)ED136*, *Df(2L)ED1378*, *Df(2L)ED1454*, *Df(2L)ED1466*, *Df(2L)ED1473*, *Df(2L)ED19*, *Df(2L)ED206*, *Df(2L)ED334*, *Df(2L)ED343*, *Df(2L)ED347*, *Df(2L)ED353*, *Df(2L)ED354*, *Df(2L)ED369*, *Df(2L)ED384*, *Df(2L)ED385*, *Df(2L)ED40*, *Df(2L)ED441*, *Df(2L)ED4559*, *Df(2L)ED4651*, *Df(2L)ED475*, *Df(2L)ED479*, *Df(2L)ED489*, *Df(2L)ED49*, *Df(2L)ED50001*, *Df(2L)ED690*, *Df(2L)ED695*, *Df(2L)ED700*, *Df(2L)ED7007*, *Df(2L)ED729*, *Df(2L)ED746*, *Df(2L)ED761*, *Df(2L)ED773*, *Df(2L)ED774*, *Df(2L)ED775*, *Df(2L)ED776*, *Df(2L)ED7762*, *Df(2L)ED777*, *Df(2L)ED778*, *Df(2L)ED779*, *Df(2L)ED784*, *Df(2L)ED7853*, *Df(2L)ED793*, *Df(2L)ED80*, *Df(2L)ED800*, *Df(2L)ED8142*, *Df(2L)ED8185*, *Df(2L)ED87*, *Df(2L)ED929*, and *Df(2L)ED94* were analyzed for both the hybrid (*w*^*1118*^/*OregonR*) and the isogenic (*w*^*1118*^) backgrounds. *Df(2L)ED1102*, *Df(2L)ED1231*, *Df(2L)ED21*, *Df(2L)ED243*, *Df(2L)ED247*, *Df(2L)ED250*, *Df(2L)ED270*, *Df(2L)ED279*, *Df(2L)ED280*, *Df(2L)ED284*, *Df(2L)ED285*, *Df(2L)ED292*, *Df(2L)ED299*, *Df(2L)ED3*, *Df(2L)ED320*, *Df(2L)ED33*, *Df(2L)ED499*, *Df(2L)ED501*, *Df(2L)ED508*, *Df(2L)ED548*, *Df(2L)ED578*, *Df(2L)ED5878*, *Df(2L)ED611*, *Df(2L)ED62*, *Df(2L)ED623*, *Df(2L)ED629*, *Df(2L)ED647*, *Df(2L)ED6569*, *Df(2L)ED678*, and *Df(2L)ED680* were analyzed only in the *w*^*1118*^ isogenic background, and *Df(2L)ED1004*, *Df(2L)ED632*, and *Df(2L)ED8186* were analyzed only in the *w*^*1118*^/*OregonR* hybrid background. *Df(2L)ED1050*, *Df(2L)ED123*, and *Df(2L)ED611* showed no clear reduction in hemizygous gene expression raising the possibility that they are not deletions, and complemented multiple mutations in genes that should be deleted. We conclude that these three lines are incorrectly identified in the collection. We removed these from the compensation analysis, although inclusion/exclusion of these *Dfs* did not significantly alter overall compensation values at the rounding levels reported here. Data from these *Dfs* is included in the GEO entries.

### RNA-Seq molecular biology

Preparation of RNA sequencing libraries for the *w*^*1118*^ isogenic background: flies were mixed with 3mm tungsten carbide beads (Qiagen, West Sussex, UK) and 500μl RNAlater solution (Life Technologies, Grand Island, NY, USA) then homogenized using a Qiagen Tissue Lyser II (Qiagen, West Sussex, UK) for 90 seconds. RNA isolation was performed using the RNeasy 96 kit (Qiagen, Valencia, CA, USA) according to the manufacturer's guide (Protocol for Isolation of Total RNA from animal cells using QIAvac96 vacuum manifold, Cat#19504) except that instead of mixing 150μl of lysis buffer and 150μl of 70% ethanol, 150μl of lysis buffer, 120μl of 100% ethanol and 30μl of the homogenate in RNAlater were used. RNA was quantified using the RiboGreen kit (Life Technology, Grand Island, NY, USA). 100ng of total RNA was used to prepare libraries based on the half-size reaction protocol from the standard Illumina guideline using the TruSeq RNA sample preparation kit v2 (set A and B, Cat # RS-122-2001 and Cat # RS-122-2002, San Diego, CA, USA). 10pg (336 samples) or 500pg (60 samples) of External RNA Controls Consortium RNAs were spiked in during the RNA fragmentation step [90]. These defined pools of RNAs were transcribed from the plasmids of the Standard Reference Material 2374 (Pool 78A and 78B), obtained from National Institute of Standards and Technology (Gaithersburg, MD, USA). RNA-Seq was carried out on a HiSeq 2000 (Illumina, San Diego, CA, USA)

Single, 3–5 day post-eclosion adult male or female flies were collected and crushed in 100μl of RNAlater (Life Technologies, Grand Island, NY). Biological samples were prepared in triplicate and crushed files were frozen at -80°C for long-term storage. A Mini-BeadBeater 96 and 1mm glass beads (Biospec Products, Bartlesville, OK) were used to homogenize flies as follows: 100μl of beads were mixed with the flies in RNAlater in 1ml Axygen 96 well plates (Corning, Union City, CA) and homogenized for 1min (repeated three times) with 2min on ice between homogenization rounds. The RNAlater solution was diluted and tissues were lysed in 600μl of RLT buffer (Qiagen, Valencia, CA) following homogenization. RNA extraction was performed using RNeasy 96 kits (Qiagen, Valencia, CA) according to the manufacturer's handbook (Protocol for Isolation of Total RNA from Animal Cells using spin technology, Cat#19504). Quant-iT RiboGreen (Life Technologies, Grand Island, NY) was used to quantify RNA before library preparation.

400ng of total RNA from the hybrid background flies in 50μl nuclease-free water were mixed with 50μl Dynabeads Oligo(dT)-25 slurry (Life Technologies, Grand Island, NY), which was pre-rinsed and 2:5 diluted with Binding Buffer (20mM Tris-HCl pH7.5, 1.0M LiCl, 2mM EDTA), and incubated at 65°C for 5min. Samples were cooled on ice for 1min. The bead-RNA complex was incubated at room temperature for 15min. Beads were collected using a magnetic stand, and rinsed with 200μl of Washing Buffer (10mM Tris-HCl pH7.5, 0.15M LiCl, 1mM EDTA) with agitation (1min at 1,000rpm with Thermomixer, Eppendorf, Hauppauge, NY). Beads were collected again and poly A^+^ RNA was eluted with Elution Buffer (10mM Tris-HCl pH7.5) at 80°C for 2min. poly A^+^ RNA was rebound to the beads by adding 50μl of Binding Buffer and rinsing as above. RNA was eluted from the beads with 16μl of Fragmentation Buffer (1:4 dilution of 5X First Strand Buffer from Protoscript II (New England BioLabs, Ipswich, MA), 500ng of random primers (Life Technologies, Grand Island, NY), and 10pg of External RNA Controls Consortium spike-ins from National Institute of Standards Technology) at 94°C for 6min. Pool 78A of Standard Reference Materials 2374 was used as a spike-in [33, 90]. Any residual beads were removed using a magnetic stand. Reverse transcription was carried out using 100 units of Protoscript II reverse transcriptase, 5mM DTT, and 625uM dNTPs (Enzymatics, Beverly, MA) with 10 units of SuperRase-in (Life Technologies, Grand Island, NY) at 25°C for 10min, 42°C for 50min, and 70°C for 15min. The DNA-RNA hybrid was cleaned up by incubating with 1.9 volumes of MagNA beads [91], and a 0.85 volume of ethanol for 15min at room temperature. The DNA-RNA-bead complexes were collected using a magnetic stand, beads were rinsed twice with 200μl of 80% ethanol and air-dried for 5min. DNA-RNA hybrids were eluted using 16μl of Elution Buffer. A strand-specific protocol for second strand synthesis was used as follows: 2.5 units of RNase H, 10 units of DNA polymerase I in 1X Blue Buffer, 10mM DTT, 0.5mM each dATP, dCTP, and dGTP (all from Enzymatics) and 1mM dUTP (ThermoFisher Scientific, Waltham, MA, 1mM final) were added to the first strand synthesis and incubated for 5 hours at 16°C. The double strand DNA products were rinsed with MagNA beads as described above. cDNA was eluted with 16μl of Elution Buffer and the same para-magnetic beads were retained for the next purification steps. DNA was end repaired using NEBNext End Repair Module (New England BioLabs) according to the manufacturer’s protocol in a 20μl volume, 1.9 volumes of XP buffer [92] added and the adenylated DNA was eluted with 10μl of Elution Buffer. 1μl of 24 differently indexed TruSeq v2 kit adapters (Illumina, San Diego, CA) were added to the eluate in 24μl ligation solution with T4 DNA ligase (Rapid) (Enzymatics, Beverly, MA) and incubated for 20 min at 20°C. The ligation was stopped by adding 8μl of 0.03M EDTA and DNA cleaned with 24μl of XP buffer as described above. DNA was eluted with 30μl Elution buffer, which was subjected to an additional clean-up with 1 volume of XP buffer. Final elution was done with 12μl of Elution Buffer. In order to remove dUTP incorporated strands of DNA, 5 units of Uracil DNA Glycosylase (New England BioLabs) were added and incubated at 37°C for 30min. PCR reactions were performed with 1.5μl of 10μM P5 and P7 primers (Sequences from Illumina were synthesized by Integrated DNA Technology, Coralville, IA). 2X KAPA HiFi HotStart DNA polymerase (KAPA biosystems, Wilmington, MA) was used in 30μl reactions. PCR parameters were 98°C for 45sec; 14 cycles of 98°C for 15sec; 60°C for 30sec; and 72°C for 30sec followed by 72°C for 5min. The amplified DNA was purified with 1 volume of MagNA beads. Libraries were quantified with Quant-iT PicoGreen (Life Technologies) and sequenced on an Illumina HiSeq 2000 (Illumina).

### RNA-Seq data analysis

Base-calling used CASAVA version 1.8.2 (Illumina, San Diego, CA, USA) and the short reads were mapped onto the *Drosophila* reference genome (Release 5, with no “chrU” and “chrUextra” scaffolds) using TopHat 2.0.11 [93] with -g 1 and -G options. An annotation file from FlyBase (5.57) was provided in the mapping process. Cufflinks 2.2.1 [94] was used to measure gene expression levels in Fragments per Kilobase per Million mapped reads (FPKM), using -G, -b, and -u parameters. FPKM values from intergenic regions were measured as described in [14]: these were used to determine expression cutoff levels as 0.6829118 for the isogenic background and 0.8140542 for the hybrid genetic background. HTseq 0.6.1p1 [95] was used with default settings but with the matching strand information to obtain raw reads counts at the gene level. Differential gene expression was assessed with “voom” in the R limma package [96] as in the referenced paper except we filtered based on intergenic FPKM values as above. We also filtered genes that have less than 1 reads from more than 50% of samples for our analysis. TMM (Trimmed Mean of M values)-scale normalization was performed using edgeR [97] to determine effective library size for the voom analysis. For differential expression analyses, replicates from one genotype were compared to those from all other genotypes in the same sex and genetic background. In determining genes with male- or female-biased expression patterns (**Fig 5E**), the same procedures as above were followed, but compared all males to all females from the isogenic genetic background. Benjamini-Hochberg multiple hypothesis correction of the *p* values was used throughout the manuscript, and adjusted *p* values less than 0.05 were called to be significant changes. FPKM values for the spike-ins were separately determined as abundant transcripts influence estimation of gene expression. In the calculation, the number of reads from both genes and spike-ins, not solely from spike-ins, was used as the denominator of FPKM to infer the lowest detectable expression levels of genes. In describing gene expression changes, expression values for genes that produce polyA^+^ mRNA in the gene model were reported, since we followed a poly-A purification protocol. For example, expression of histone transcripts were detected, but the values were highly variable between libraries due to their lack of poly-A tails, and essentially followed the values of residual rRNAs in the sequencing libraries [98]. Expression results presented were robust to normalization methods used in different bioinformatics tools (FPKM, DESeq, and TMM). The Expectation-Maximization method was used to identification of the fully compensated group of genes in Fig 4 using “mclust” package in R [99]. In the analysis, two ellipsoidal distributions of equal orientation, “EEE”, were used as models for the clustering algorithm.

TopHat was used to identify fusion transcripts. Potential fusion transcripts that have at least 15 bp long anchor sequences were surveyed. For intra-chromosomal fusion events, we investigated potential fusion transcripts with more than 683 bp distances between juxtaposed regions based on the shortest length of *Dfs* (—fusion-anchor-length 15 and—fusion-min-dist 683 parameters).

**Fig 10:**
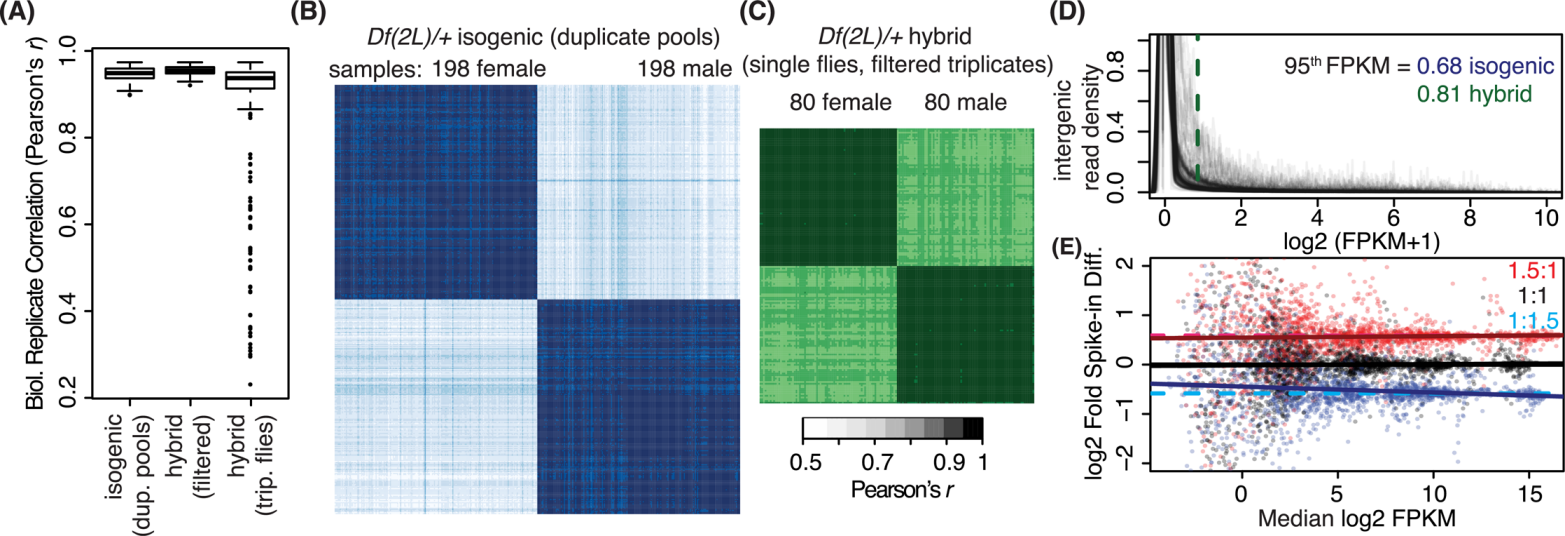
Replicate reproducibility in RNA-Seq results and determination of gene expression cutoff. A) Box plots that display distribution of Pearson’s correlation coefficient (*r*) between experimental replicates. The second and third boxes demonstrate *r* values between replicates from the hybrid genetic background, where the second one is after filtering at *r* < 0.9. B, C) All sample-to-all sample pairwise comparisons. The opacity of color indicates Pearson’s *r* as illustrated in the key. D) RNA-Seq signals from intergenic regions are collectively overlaid as density plots across signal in FPKM. Median of the top 95 percentile in the distribution is indicated (dotted lines). E) Signals from ERCC spike-ins. A pair of spike-in sets is composed with known amount of RNA species that have either 1:1 or 1:1.5 ratios between libraries. In the plot, the black horizontal line indicates expected fold difference from the subpool of spike-in RNAs that have 1:1 ratio between the pair. Red and Blue horizontal line show the expected fold differences from subpools of external RNA that have 1:1.5 ratios. Red, Black, and Blue points indicate the actually measured ratio of each spike-in RNA species after normalization, at different abundances of gene expression (x-axis). Dot lines are trend lines from linear modeling.

The RNA-Seq data showed excellent biological replicate profiles (Pearson's *r* >0.9) in all 396 isogenic samples (**Fig 10A**). However, 10% outliers were observed in the single fly profiles and all samples where Pearson's *r* <0.9 were removed, the two duplicates with the highest correlation were used. The sexual identify of a sample is self-reported in the expression profile (**Fig 10B and 10C**). Detection of low-level gene expression is complicated by the contributions of noise, which vary between libraries. To mitigate this problem reads from intergenic regions were measured (trimmed to account for variation in transcription start and stop sites) and the 95th percentile determined as a low expression cutoff (**Fig 10D**). While some of this intergenic expression may be due to strain-specific transcripts or un-annotated genes, much is likely to be due to noise such as ectopic Pol-II initiation, inclusion of contaminating genomic DNA, sequencing, and/or mapping errors. Ratios and data compression measurements are critical for dosage compensation analysis. Pools of ERCC controls were used in each sample library to produce 1.5:1, 1:1, and 1:1.5 ratios across a > 2^15^ input concentration range (**Fig 10E**). Ratio measurements show a clear increase in scatter when input was low. However, even at very low input, there was only modest compression, and no evidence of compression in the useful range for this work.

### Data access

The gene expression profiles generated in this study are available in GEO with accession numbers of GSE61509 (isogenic genetic background) and GSE73920 (hybrid genetic background).

## Supporting Information

S1 File Gene expression in *Df/+* flies. Tab-limited text files contain log2 gene-level expression relative to the global reference. Genes are by Flybase IDs. Genotypes are abbreviated by removing the suffix *Df(2L)*.
(ZIP)

S2 File Significant differential gene expression in *Df* lines. Adjusted *p* values for gene expression in a tab-limited text format. Formated as in S1 File.
(ZIP)

S3 File Propagation of gene expression changes in a network space. Movie displaying gene expression changes in the isogenic background (left), and hybrid genetic background (right) superimposed on an integrated gene network model [48]. Genes up-regulated (red) or down-regulated (green) are shown. Genotypes, and sexes are indicated.
(MP4)
